# Supplementary material for: Cryopreservation effect on DNA methylation profile in rainbow trout spermatozoa
Source: Sci Rep. 2023 Nov 3;13:19029. doi: 10.1038/s41598-023-44803-2 (PMC10624875; doi:10.1038/s41598-023-44803-2)
Supplement: Supplementary file 1 — Supplementary Table 1. [file 41598_2023_44803_MOESM1_ESM.docx]

| **Regions** | **Nber of DMCs DMSO** | **Nber of DMCs MeOH** | **Nber of DMCs Gly** | **Genomic feature** | **Gene** | **Gene ID** |
| --- | --- | --- | --- | --- | --- | --- |
| **1 : 77 284 194 -**  **1 : 77 284 341** | NA | 0 | 7 | Other | NA | NA |
| **2 : 51 809 891 -**  **2 : 51 809 918** | 0 | 2 | 0 | Other | NA | NA |
| **2 : 55 059 488 -**  **2 : 55 059 512** | NA | 2 | NA | Other | NA | NA |
| **2 : 84 969 754 - 2 : 84 969 757** | 2 | 0 | NA | Introns | NA | ENSOMYG00000033911 |
| **3 : 18 143 279 -**  **3 : 18 143 295** | 0 | 2 | 0 | Other | NA | NA |
| **3 : 28 999 827 -**  **3 : 28 999 911** | 0 | 0 | 4 | Promoter_5kb | znf385d | ENSOMYG00000007065 |
| **3 : 38 641 242 -**  **3 : 38 641 260** | 0 | 0 | 2 | Introns; intron1 | ptprub | ENSOMYG00000000944 |
| **5 : 87 773 628 -**  **5 : 87 773 708** | 0 | 0 | 6 | Other | NA | NA |
| **6 : 27 006 900 - 6 : 27 006 938** | 3 | 0 | NA | Promoter _5kb | ebf2 | ENSOMYG00000007222 |
| **6 : 63 230 513 -**  **6 : 63 230 563** | 0 | 3 | 0 | Introns | adam10a | ENSOMYG00000010593 |
| **7 : 66 339 321 -**  **7 : 66 339 343** | NA | 2 | NA | Other | NA | NA |
| **8 : 49 417 188 -**  **8 : 49 417 201** | NA | 0 | 2 | Other | NA | NA |
| **8 : 62 178 762 - 8 : 62 178 843** | 6 | 0 | NA | Other | NA | NA |
| **8 : 79 446 507 -**  **8 : 79 446 591** | 2 | 2 | 4 | Other | NA | NA |
| **8 : 79 446 729 - 8 : 79 446 753** | 2 | 1 | 0 | Other | NA | NA |
| **8 : 79 446 753 -**  **8 : 79 446 760** | 1 | 2 | 0 | Other | NA | NA |
| **9 : 13 905 316 -**  **9 : 13 905 359** | 0 | 0 | 2 | Exons | NA | ENSOMYG00000012701 |
| **9 : 23 506 605 -**  **9 : 23 506 613** | NA | 2 | 0 | Other | NA | NA |
| **9 : 40 751 199 -**  **9 : 40 751 319** | 0 | 3 | 0 | Promoter_5kb | NA | ENSOMYG00000007596 |
| **9 : 65 922 544 -**  **9 : 65 922 597** | 0 | 3 | 0 | Other | NA | NA |
| **10 : 58 822 172 - 10 : 58 822 181** | 2 | NA | NA | Introns; intron 1 | NA | ENSOMYG00000026688; ENSOMYG00000026627; ENSOMYG00000026688; ENSOMYG00000026627 |
| **11 : 41 239 948 - 11 : 41 239 982** | NA | 0 | 2 | Other | NA | NA |
| **11 : 75 710 382 - 11 : 75 710 453** | NA | 3 | 0 | Other | NA | NA |
| **12 : 87 125 997 - 12 : 87 126 003** | 2 | NA | NA | Other | NA | NA |
| **14 : 50 325 080 - 14 : 50 325 136** | 0 | 2 | 0 | Introns; intron1 | pcdhb | ENSOMYG00000014270 |
| **16 : 13 278 338 - 16 : 13 278 342** | NA | 2 | NA | Introns | SLC39A11 | ENSOMYG00000033173 |
| **17 : 57 694 790 - 17 : 57 694 858** | 0 | 2 | 0 | Introns | cdh4 | ENSOMYG00000044618 |
| **18 : 26 437 868 - 18 : 26 437 930** | NA | NA | 7 | Promoter_5kb | OSBPL10 | ENSOMYG00000025071 |
| **18 : 28 780 753 - 18 : 28 780 850** | 5 | 0 | 0 | Other | NA | NA |
| **18 : 53 775 926 - 18 : 53 775 987** | 0 | 2 | NA | Other | NA | NA |
| **19 : 14 572 357 - 19 : 14 572 390** | 0 | 0 | 2 | Exons; introns; intron1 | ndufs8a | ENSOMYG00000005413 |
| **19 : 43 252 495 - 19 : 43 252 511** | 0 | 0 | 2 | Promoter_5kb | Ick | ENSOMYG00000032229 |
| **19 : 45 634 077 - 19 : 45 634 080** | NA | 2 | NA | Introns | col12a1a | ENSOMYG00000006853 |
| **20 : 20 273 -**  **20 : 20 301** | 0 | 3 | 1 | Downstre-am | 5_8S  _rRNA | ENSOMYG00000008170 |
| **20 : 3 482 719 - 20 : 3 482 759** | 3 | NA | 0 | Introns | cdhr1 | ENSOMYG00000009577 |
| **20 : 4 582 942 - 20 : 4 582 970** | 0 | 1 | 2 | Other | NA | NA |
| **20 : 13 286 267 - 20 : 13 286 314** | 2 | 0 | 1 | Promoter _5kb | p4ha1a | ENSOMYG00000043711 |
| **20 : 24 528 122 - 20 : 24 528 126** | 0 | 2 | 0 | Other | NA | NA |
| **20 : 30 738 907 - 20 : 738 987** | 0 | 0 | 3 | Introns | khdrbs2 | ENSOMYG00000043413 |
| **21 : 51 651 818 - 21 : 51 651 870** | 2 | 0 | 1 | Other | NA | NA |
| **23: 47 115 194 - 23: 47 115 229** | 2 | 0 | 0 | Other | NA | NA |
| **25 : 19 063 744 - 25 : 19 063 823** | 0 | 4 | NA | Introns; intron1 | slc26a2 | ENSOMYG00000017229 |
| **25 : 82 566 468 - 25 : 82 566 493** | NA | 1 | 2 | Introns | smoc1 | ENSOMYG00000037447 |
| **27 : 391 849 -**  **27 : 391 854** | 2 | NA | NA | Other | NA | NA |
| **29 : 15 383 450 - 29 : 15 383 576** | NA | 11 | 0 | Exons; exon1 | NA | ENSOMYG00000042159 |
| **MSJN01 009 823.1 : 484 - MSJN01009823.1 : 569** | 3 | 2 | 0 | Other | NA | NA |
| **MSJN01 009 823.1 : 538 - MSJN01009823.1 : 569** | 2 | 6 | 0 | Other | NA | NA |
| **MSJN01 011 709.1 : 7 185 - MSJN01011709.1 : 7 215** | 0 | 2 | 0 | Other | NA | NA |
| **MSJN01 019 798.1 : 257 543 - MSJN01019798.1 : 257 574** | NA | 0 | 2 | Introns; Introns1 | NA | ENSOMYG00000040507 |
| **MSJN01 041 575.1 : 20 760 - MSJN01041575.1 : 20 774** | 0 | 2 | NA | Other | NA | NA |
| **MSJN01 051 995.1 : 1 354 - MSJN01051995.1 : 1404** | 0 | 3 | 0 | Other | NA | NA |
| **MSJN01 053 504.1 : 13 293 - MSJN01053504.1 : 13 312** | 1 | 2 | NA | Other | NA | NA |
| **MSJN01 053 808.1 : 39 504 - MSJN01053808.1 : 39 747** | 0 | 0 | 8 | Introns | NA | ENSOMYG00000030867 |
| **MSJN01 064 445.1 : 1089 - MSJN01064445.1 : 1113** | 0 | 3 | 0 | Other | NA | NA |
| **MSJN01 064 445.1 : 1 194 - MSJN01064445.1 : 1261** | 2 | 10 | 0 | Other | NA | NA |
| **MSJN01 064 445.1 : 1 227 - MSJN01064445.1 : 1 247** | 3 | 2 | 0 | Other | NA | NA |
| **MSJN01 067 055.1 : 47 598 - MSJN01067055.1 : 47624** | NA | 5 | 0 | Other | NA | NA |
| **MSJN01 068 470.1 : 831 - MSJN01068470.1 : 873** | 0 | 2 | 0 | Exons; exon1; promoter_5kb | U2 for promote-r_5kb | ENSOMYG00000041560; ENSOMYG00000041563 for promoter_5kb |
| **MSJN01 119 667.1 : 20 426 - MSJN01119667.1 : 20 474** | 0 | 2 | 0 | Other | NA | NA |
| **MT : 6 657 -**  **MT : 6754** | 0 | 2 | 0 | * : see legend | | |

**Supplementary Table S1: List of the potentially sensitive regions according to the cryoprotectant** The sequenced regions which displayed at least 2 DMCs (differentially methylated cytosines) between fresh and cryopreserved samples within 100 b (read size) were included in this list. Gly: glycerol. The “Regions” column indicates the chromosome number and the region coordinates on the reference genome. Gene : name from the ensembl database (version 104) according to the reference genome (Omyk_1.0 genome version). Promoter 5 kb: region 5 kb upstream of the transcription starting site; Exon 1: first exon; Downstream: region 1 kb downstream of the transcription end site; Other: intergenic regions; NA: not available. *the 2 DMCs belonged to the exon1 of *cox1*, downstream of *nd2*, promoter 5 kb of *cox2*, promoter 5kb of *atp8*, promoter 5kb of *atp6*, promoter 5kb of *cox3*, promoter 5kb of *nd3*, promoter 5kb of *nd4l*, promoter 5kb of *nd4.*
